# Context and intricacies in the evolution of master of public health programmes in post-apartheid South Africa

**DOI:** 10.3389/fpubh.2026.1882277

**Published:** 2026-10-14

**Authors:** Thembelihle Dlungwane, Virginia Zweigenthal, Laetitia Rispel

**Affiliations:** 1 Discipline of Public Health Medicine, College of Health Sciences, Howard College Campus, University of KwaZulu-Natal, Glenwood, South Africa; 2 Division of Public Health Medicine, School of Public Health, Faculty of Health Sciences, University of Cape Town, Cape Town, South Africa; 3 South African Research Chairs Initiative (SARChI), School of Public Health, University of Witwatersrand, Johannesburg, South Africa

**Keywords:** context and intricacies, evolution of MPH programmes, post-apartheid South Africa, postgraduate medical education, public health education

## Abstract

**Introduction:**

Globally, Master of Public Health (MPH) programmes provide essential skills in epidemiology, health policy, and management and prepare graduates to tackle complex health challenges across the lifespan, and to strengthen health systems. This study is a critical analysis of the evolution of MPH programmes in post-apartheid South Africa and the complex interplay between historical legacies, health system transformation, and shifting national and global public health priorities.

**Methods:**

An exploratory, descriptive qualitative study combined in-depth interviews with key informants and a review of relevant documents on the evolution of MPH programmes. The interviews explored stakeholder perspectives on the evolution, development and transformation of MPH programmes, while the document review examined the historical development and evolution of MPH and postgraduate public health training in South Africa. Data were analysed thematically.

**Results:**

MPH programmes emerged in the context of profound socio-political change following South Africa’s democratic transition in 1994. These programmes were developed to address health workforce gaps, redress apartheid-era inequities, and cultivate a multidisciplinary cadre of health worker equipped to respond to the country’s growing burden of disease and determinants of health. However, MPH programmes have been shaped by the tension between global public health standards and local contextual realities, variation in institutional capacity in different universities, and the need to balance academic rigour with practical relevance for a diverse health workforce. Notwithstanding several innovations, insufficient government investment and the lack of career pathways for MPH graduates remain unresolved issues.

**Conclusion:**

MPH programmes in South Africa were shaped by contextual drivers and institutional dynamics, with ongoing developments linked to national efforts to address inequities inherited from apartheid and to build a multidisciplinary workforce capable of implementing population-based health strategies. Future research should compare public health curricula across South African institutions.

## Introduction

The discipline of public health, rooted in the prevention of disease and the promotion and protection of health through organised societal efforts, has evolved to address the social, political, economic, and environmental determinants of health (1, 2). In the 19th century, its formalisation was driven by germ theory, sanitation reforms, and the establishment of major institutions like the US Centres for Disease Control and Prevention (CDC) and the Pasteur Institute (3–6). These developments laid the groundwork for modern health public practices and, later, for the development of education programmes, including the Master of Public Health (4).

Country contexts have influenced the development of public health training programmes. In several countries, public health training was initially reserved for specialised medical doctors, limiting opportunities for other health professionals or graduates from other disciplines (7, 8). After World War 2, the growing complexity of health systems created a need for multidisciplinary expertise, and public health education began to move beyond the training of medical practitioners (8–11). First established in the 1930s in the United States of America (USA) to develop public health services, Master of Public Health (MPH) programmes were designed to play a critical role in preparing professionals to address complex challenges and analyse data, identify trends, develop evidence-based interventions, and effectively communicate with diverse stakeholders to promote the health of populations (12–14).

In low and middle-income countries (LMICs), postgraduate public health training programmes historically tended to replicate the models of high-income countries and/or former colonial powers (7, 15). However, the 1978 Alma-Ata Declaration on Primary Health Care (PHC) was a critical milestone for the re-orientation and expansion of public health programmes in LMICs (16). Schools of Public Health in LMICs have contributed to the development of cadres of competent and well-motivated public health professionals who ask questions that address fundamental health determinants, seek solutions as agents of change within their mandates, provide specific services and serve as advocates for multilevel partnerships (16).

South Africa’s 1918–1919 influenza epidemic, which resulted in an estimated 500,000 deaths, exposed its fragmented and racially segregated public health system and prompted the enactment of the landmark Public Health Act of 1919, creating a central authority to coordinate services (17, 18). However, the benefits of this legislation were unevenly realised, as its implementation was constrained by the racially discriminatory policies that shaped health governance during that period (17). In the decades of white minority and later, apartheid rule, there were initiatives that challenged fragmented and inequitable access to health care (19). In the 1980s, in response to the growing levels of political repression, there was the emergence of a progressive health sector, comprising a movement of health professionals, academics, and activists committed to the pursuit of health equity, social justice, and structural reform (20, 21). This led to the institutionalisation of public health as an academic discipline, where research and teaching were entrenched, as a strategic tool for achieving equity and social justice in the post-apartheid era.

The original intention was to develop MPH programmes in geographic regions, and for universities to collaborate and offer courses jointly so as to capitalise on scarce public health academics and teachers. However, this did not materialise. Over the years, the number of MPH programmes offered in SA has increased from three in 1990 to eleven in 2022. In terms of geographical location, four programmes are situated in Gauteng, two in the Western Cape, two in the Eastern Cape, two in Limpopo and one in KwaZulu-Natal (22). Most programmes are offered part-time and cater for students from multidisciplinary backgrounds compared to its the inception, when it was dominated by medical doctors. Most programmes provide a wide range of concentrations, and comprise coursework and a research component (22).

Since 1994, South Africa has undergone profound political, social, and institutional change, accompanied by major shifts in population health priorities, health governance, and equity-oriented policy frameworks (23). These shifts have shaped the purpose, content, and positioning of public health training. The evolution of MPH programmes in post-apartheid South Africa represents a critical yet under-examined dimension of the country’s broader transformation in health systems, human resources for health, and higher education. This paper begins to respond to this knowledge gap by providing a contextualised analysis of how MPH programmes have developed, diversified, and adapted over time.

The novelty of the paper is its focus on the post-apartheid landscape in South Africa, including the HIV & AIDS epidemic in the early years of democracy, rising non-communicable diseases, persistent health inequities, decentralisation and transformation of the health system, and renewed emphasis on primary health care (PHC) and Universal Health Coverage (UHC) (23). The evolution of MPH programmes has been shaped by these dynamics, but not in uniform or linear ways. By examining the intricacies of MPH programme evolution from the perspectives of pioneering academics and historical material, the paper highlights how context, institutional histories, and professional debates have interacted to shape public health education.

MPH programme evolution in South Africa provides a useful case study for understanding how public health education adapts in settings marked by historical injustice, policy ambition, and competing global and local influences. This has relevance for other low- and middle-income countries undergoing social transition, education reform, or health system restructuring.

## Methods

An exploratory, descriptive qualitative study design was used, which combined in-depth interviews with key informants (KIs) and an analysis of relevant documents that describe the evolution of the MPH programmes. Each component is described below.

### Key informants interviews

The purpose of the key informant interviews (KIIs) was to explore the perspectives of stakeholders on the evolution of the MPH programmes. We developed a map of all the potential stakeholders, targeting knowledgeable individuals able to provide in-depth and rich information on the evolution of MPH programmes. These individuals played a central role in the motivation or initiation of the programmes (considered pioneers of postgraduate public health training) and/or who continue to be involved in the MPH programmes at the various training institutions in South Africa. We compiled a list of six potential key informants.

Additionally, we used snowballing technique, by asking key informants who agreed to be interviewed to recommend other potential key informants for interviews. We developed a semi-structured interview guide based on the aims and objectives of the study. The interview guide consisted of open-ended questions, and prompts were used. Topics included the background to and history of the MPH programme evolution, the health, political and global contexts that influenced the programme development, their perspectives on MPH training in SA, their involvement in curriculum design, progress and challenges. The interview schedule was piloted with two academics who have similar profiles to the key informants. Based on the feedback and observations during the pilot, no changes were necessary. The information from the pilot interviews was excluded from the analysis.

Potential key informants were contacted via email or telephone for interviews. Following verbal agreements, each consenting informant was provided electronically with the study information sheet, consent forms for the interview and digital recording, and the interview guide. The principal researcher (TD) conducted virtual in-depth interviews using the Zoom Platform between April 2022 and October 2022. At the beginning of each interview, verbal consent and permission to record the interview were obtained. The background and the purpose of the study were explained. All the interviews were conducted in English. The interviews took 45–60 min.

### Document review

The purpose of the document review was to explore the development and evolution of MPH or postgraduate public health training programmes in South Africa. We focused on the period from 1990 onwards, corresponding roughly with the release of Nelson Mandela in February 1990 until 2024, when South Africa celebrated 30 years of democracy. We assumed that these are the primary documents that shaped the development and evolution of the MPH training programmes. The document review took place during 2022 and 2024. We applied the READ approach - a systematic process to guide document analysis - to the document review (24). This involves a systematic four step procedure in reviewing documents: (1) Ready your materials, (2) Extract data, (3) Analyse data and (4) Distil your findings (24).

Our first step was to source our materials for the document review and analysis by searching for published and grey literature through Google Scholar and PubMed published from 1993 to 2022 (see Supplementary material). Keywords used to search the published literature included: ‘Master of Public Health Programmes’, ‘Schools of Public Health’, ‘South Africa’, ‘Postgraduate Public Health Training’. All articles and documents were written in English.

We identified 25 potential documents for inclusion in the review. Following this initial identification of legislation and policies, the principal investigator (TD) then embarked on filtering through the documents to include those specifically related to MPH programme evolution (criteria for inclusion). Of those 25 potential documents identified during the initial search, 18 documents met the criteria for inclusion. A full list of all documents initially identified are included in a Supplementary material.

### Data analysis

The analysis followed several steps. All interview transcripts and documents were read repeatedly to achieve familiarisation with the data. During this stage, preliminary notes and analytic memos were developed to capture initial observations. Firstly, the PI extracted the relevant codes from the 18 documents and entered the data into an MS Excel spreadsheet/ grid. This process was guided by our research question: how did the MPH programme evolve in democratic South Africa?

Secondly, each interview was audio recorded and transcribed verbatim in preparation for analysis. The transcriptions were done by a professional transcriber and verified by the authors. The researcher (TD) read the written transcript of each participant’s interview numerous times to gain a full understanding of the phenomenon and then to identify codes. A thematic analysis was implemented. The respondent (R) quotations reflecting notions are given as R1–R6.

Thirdly, the coding framework was applied to both datasets. Interview transcripts and documentary evidence were coded separately in the initial stage to preserve the integrity of each data source. Once coding was completed, the codes were compared across the two datasets to identify areas of convergence, complementarity, and divergence. Integration of the document review and interview findings occurred through triangulation, with themes emerging from interviews being examined alongside documentary evidence. In cases where interview data supported information found in documents, the findings were interpreted as convergent evidence. Where interview narratives provided additional detail or contextual explanations beyond what was documented, the two sources were treated as complementary. To enhance credibility, emerging themes and subthemes were discussed among members of the research team. All team members independently reviewed and discussed the identified codes, with discrepancies resolved through discussion until consensus was reached. This was an iterative process over a period of 2 years.

### Ethical considerations

Ethical approval was granted by the University of KwaZulu-Natal Human and Social Sciences Research Ethics Committee (HSSREC/00003710/2021). All who agreed to participate in the study signed informed consent. Participation was voluntary, and participants were informed that they could withdraw without prejudice. The transcripts were labelled with unique numbers to ensure confidentiality.

## Results

The key informants who participated in this study were recruited from academics who were involved in the establishment of the Schools of Public Health in different regions within South Africa. They played a significant role in the training of public health professionals, and they had between 30 and 40 years of experience in the field of public health. In Table 1, of the six key informants who participated in the study, five were physicians.

**Table 1:** Profile of key informants.

Participant	Role in public health training	Years in public health	Current position	Gender
R1	Head of Department	30–40	Emeritus Public Health Specialist and Professor	Male
R2	Head of Department	30–40	Public Health Specialist and Professor	Male
R3	Senior Academic	20–30	Public Health Researcher and Professor	Female
R4	Head of Department	30–40	Public Health Specialist and Professor	Male
R5	Senior Academic	30–40	Public Health Specialist and Senior Academic	Male
R6	Senior Academic	30–40	Emeritus Public Health Specialist and Professor	Male

Table 2 shows the themes that emerged from the interviews and document analysis. The first theme, history and context, highlights that the evolution of MPH programmes cannot be viewed outside the broader socio-political environment. The second theme captures the driving forces of MPH programme development, but also the progress and achievements. The third theme covers the contestations and intricacies of the original intention of developing regional schools of public health, what happened in practice, and the ongoing discourse of MPH professionals vs. Public Health Medicine specialists. Although the themes overlap, they are presented separately for the sake of clarity.

**Table 2:** Themes and emerging sub-themes.

Theme and explanation	Sub-themes
*History and context* The evolution of MPH programmes seen within the broader socio-political environment	Progressive/anti-apartheid health organisations Transition to democracy Health Plan of the African National Congress Burden of disease Health system transformation Crisis in and demand for human resources for health
*Activism and leadership* The driving forces of the MPH programme development, its progress and achievements	Role of activism Strengths and innovations Number of Schools of Public Health Multi-disciplinarity Diversity in curricula and delivery methods Influence and impact of Schools and graduates
*Contestations and intricacies* The original intention of developing regional schools of public health, what happened in practice, the ongoing discourse of MPH professionals vs. Public health medicine specialists.	Public health medicine vs. MPH degree Thusano, National School of Public Health, independent schools and degrees MPH at universities with medical schools vs. those without Insufficient collaboration across schools

### History and context

The evolution of MPH programmes cannot be viewed outside the broader socio-political environment, the changes that were taking place in the country and the new health reforms that were proposed. The 1994 Reconstruction and Development Programme (RDP) was a socio-economic policy framework implemented by the ANC-led government in South Africa to address the massive inequalities, poverty, and structural economic problems created by apartheid. In health, the RDP underscored the importance of health equity, addressing the social and environmental issues that affect population health, an integrated and inclusive national health system the implementation of the Primary Health Care Approach.

The dawn of democracy in 1994 signalled a new era for public health with the adoption of a rights-based approach to healthcare (25). The post-apartheid government prioritised the transformation of the health system to address inequities and ensure universal access to quality healthcare services (26, 27). Several initiatives came into effect, and there was a call to shift from a curative-based healthcare system to promotive and preventative health that focuses on the appropriate levels of care depending on the burden of disease and disease severity.

There was a need to move to a more comprehensive approach to health and its determinants, such as environmental issues and community-responsive services. The 1997 White Paper on the Transformation for the Health System set the policy framework to ensure that the primary healthcare approach was the foundation of the health system (28). The introduction of progressive health legislation, such as the 2003 National Health Act enshrined patients’ rights and the shift from an individual healthcare approach to a broader public health perspective to and mechanisms to achieve population health outcomes (29).

The development of postgraduate public health training programmes in SA was also influenced by a growing recognition of the need for health professionals with skills in epidemiology, biostatistics, and health management to address challenges from a broader population health perspective (30, 31). Hence, most MPH programmes prioritised these skills. As one key informant remarked:

*All the students are exposed to the core modules which are epidemiology, biostatistics, health promotion and health systems and policy* [R3]

The MPH also became the avenue to orientate health managers to a public health approach that viewed health facilities as part of a functioning health system.

*One issue was that medical superintendents and matrons ran the hospitals and, after 1994, some of them also assumed the role of hospital managers. Most did not have prior academic exposure to health service management, so they were subsidised to do an MPH in health service management.* [R5]

The new health reforms acknowledged that the transformation of the health system would require substantial training and development for managers and all health professionals in the public health system (28). This transformation influenced healthcare professional training, leading to MPH curricula that adopted a holistic view of health and its determinants, encompassing key environmental, social, and community-based dimensions. It prepared personnel to address critical determinants of health.

*The White Paper specifically says training should focus more on preventing disease and promoting health. So, I do not know the amount of health promotion the various programmes do, but I know that everyone has at least some health promotion. The focus area was the … community-oriented primary health care, engraved in the 1997 White Paper of government, promoting the introduction of health promotion and disease prevention in healthcare sciences, so that influenced us* [R3]

*‘Anyone interested in the anti-apartheid movement saw this coming… And so, the MPH opened a set of opportunities to help prepare the public health sector for the new South Africa. If the country were going to improve on health, it would need to talk about water, sanitation, employment, safety, redistribution of wealth and access to resources which were not available to many people; it would have to talk about community engagement, all that stuff wasn’t part of the curriculum.’* [R4]

### Activism and leadership

Activism, leadership in government and academic institutions played a key role in shaping public health education in South Africa, particularly during and after the transition to democracy. Rooted in the country’s history of social justice movements, activism resulted in the development of health organisations and advocacy. These individuals and organisation influenced the training of health professionals towards equity, human rights, and community empowerment (32, 33).

*The group of us came together in 1981 to form an alternative Medical Association, the National Medical and Dental Association NAMDA. NAMDA played a very important role, spearheading a key role in exposing the relationship between apartheid and health. We had numerous conferences and branches all over.* [R1]

*The debates and focus on policy modules were to some extent influenced by the broader philosophical and political movements in the country.* [R6]

Informants noted that most programmes collaborated closely with the transformed Department of Health, and were supported by top leadership of universities. The government to send managers to be trained in epidemiology, health policy and management in line with the health reforms that were in place post-1994, which enabled them to be change agents in the health system.

*We had a very strong and excellent collaboration with the Department of Health [with both national ministers from 1994–2014], and the collaboration was done in two ways. We train their staff so as to upskill them in epidemiology, management and health policy. There was a very synergistic relationship between the training that we were giving and the fact that people were able to go back and positively impact beneficiaries by transforming the health system*. [R1]

*I was appointed head of the Department of Community Health. And I had the strong support from management, the Vice Rector, and later, the Rector to set up a school of public health and to run the programme.* [R4]

Furthermore, the need for a critical mass of health professionals with broad public health knowledge was highlighted as one of the driving forces behind the evolution of MPH programmes in South Africa. The gap was seen when health professionals with clinical backgrounds were expected to take on health management positions. They had not been exposed to any training in health service management to prepare them for those roles. In addition to promotive and preventive health skills, informants identified skills and training gaps, such as epidemiology, research, policy and management. This included facility management and management at the national and provincial levels. This included reviewing health data and evidence to inform strategic direction.

*We structured the MPH programme, and it took a lot of service people to upskill them. So, the first task we had was to update the existing clinicians, environmental health people, and nursing people and give them public health exposure training in the foundation, which is epidemiology and biostatistics, health promotion, management and control of infectious and communicable and non-communicable diseases. And then a core of MPH and public health was management.* [R5]

*We ran a major programme and trained hundreds of people in the national and the other provincial health departments on health policy and management.* [R6]

Most academics involved in the inception of MPH programmes had their public health training in the United Kingdom or the United States of America. The Global North heavily influenced the postgraduate public health curriculum, and that was reflected in the programme structure and the modules offered at the inception.

*I studied and qualified in community health. I did a fellowship in the US and came back in 1990. There was a wave which affected all the universities, and I’m not sure where the wave was coming from, presumably from developments overseas and people returning. There was a feeling that we should be looking for a broader offering than just our registrars (medical officers).* [R2]

In one institution, MPH modules were first offered through distance education at George Washington University in the USA and Hebrew University in Israel. Over time, the development of expertise and being attentive to the South African context, this was discontinued and a local curriculum was taught locally.

*We partnered with two international schools of public health so the overseas academics will come to South Africa for face-to-face summer school sessions and continue with students online.* [R3]

*When I joined the school then, we started developing our curriculum, and two years later, that’s when we started teaching the bulk of the curriculum using our South African curriculum.* [R3]

### Contestations and intricacies

This theme covers the contestations and intricacies of the original intention of developing regional schools of public health, what happened in practice, and the ongoing discourse in postgraduate public health training. Postgraduate public health training in SA originated in medical schools, within departments of community medicine, and was designed as specialist training for medical doctors (12, 34).

After the promulgation of the 1919 Public Health Act, many universities offered Diplomas in Public Health, which were designated for medical officers (35, 36). At the time medical professionals dominated the public health field, and the qualification did not cater to professionals from other disciplines. These diplomas did not sufficiently prepare graduates for complex public health practice and so training in community health was developed that allowed specialist registration with the country’s medical council. This remains open to only medical doctors, who train full-time for 4 years. They obtain theoretical and practical training using an apprenticeship model. The qualification, conferred by the Colleges of Medicine, is now named Public Health Medicine.

*We started off by offering the diploma for doctors, so all of the Diploma students were medical superintendents or medical managers.* [R5]

*We started off with the speciality degree called the Fellowship of the Faculty of Community Health, which was a four-year public health medicine degree, and it was predominantly only for doctors.’* [R1]

Although most public health schools were part of the medical school system, one school was created independently of the medical faculty. Unlike schools that were part of medical schools employing specialists who fell under hospital or health establishments, this school did not benefit from staff being funded in this way.

*There was a regional development where they were trying to get the three universities in the Western Cape into the same structure. And that did not work out that way. So, in the end, each university carried on along its own line. Two institutions developed their own MPHs. They [UWC] created a free-standing School of School of Public Health. But it’s unique in that it was not based in the medical school; all the others were based largely in the faculties of medicine.* [R6]

The need to develop Schools of Public Health was discussed in 1990, as reflected in the Tertiary Education Linkages Project (37). Subsequently, three regional Schools of Public Health, namely the Transvaal School of Public Health, the Eastern Seaboard Association of Tertiary Institutions and the Western Cape School of Public Health, were proposed (37). In addition, a National School of Public Health was established at the Medical University of South Africa (37). The primary purpose was to form a joint undertaking between all the relevant regional institutions and to maximise the skills and resources by using shared teaching and learning platforms (37).

The Transvaal School of Public Health, later known as the Thusano School of Public Health (TSPH), was founded in 1991 (37). The partners in the TSPH were Potchefstroom University and the Universities of the North, Pretoria, Venda and Witwatersrand (37). The partnership aimed to strengthen public health training in the northern regions of SA. The idea was that the degree of MPH was to be offered by TSPH and conferred by the Universities of Pretoria and Witwatersrand (37–39). The partnerships could not be sustained due to territorial and institutional bureaucracies. Several issues were not considered at the beginning of this initiative including subsidies, as well as the allocation of funding for research and student supervision.

*We tried to get to the Transvaal School of Public Health off the ground. We relabelled it Thusano because Transvaal was the old terminology. And the effort broke down. I stopped trying to get six or seven institutions to work together because it’s so difficult unless there is a new ministerial directive to do so. So, we could not get the Transvaal school to go, so it’s split up between universities. So, Pretoria did its own, and Wits then followed. And then since then, Schools of Public Health have become a thing.* [R4]

In the Western Cape province, the initial idea was to develop a single regional school of public health The intention was to bring together the three universities: the University of Cape Town, Stellenbosch University and the University of Western Cape (37). Efforts to get these institutions to work together did not bear fruit. Cape Town and Stellenbosch universities already had active Departments of Community Health affiliated with medical schools with strong leadership.

*Everybody was doing their own thing in Cape Town; it was links between UCT and UWC. In fact, we ran some core courses with them. There was a lot of politics in Cape Town. Stellenbosch, for example, never established an MPH. There was a lot of politics, and that also influenced things. That was real competition for resources being close to each other.* [R2]

The National School of Public Health (NSPH) was established with the support of the Department of Health in 1998 in the former Medical University of Southern Africa (Medunsa), Pretoria (30). The NSPH intended to have a national character by developing a comprehensive distance-learning approach to public health education, which would become the educational gateway for various health professionals who wanted to further their training in postgraduate public health (37). Although a dean was appointed, there were no staff or formal relationships or collaborations with other regional schools (37). The NSPH was situated on the Medunsa campus, which had a Department of Community Health, but there was no interaction or collaboration between the two.

*The National School of Public Health was started by one of the epidemiologists. He felt that he should set up a School of Public Health in a historically black institution, as opposed to Pretoria or Western Cape, although Western Cape is also a historically black institution that he would set up. So, the national school came into competition. There was never any effort to connect the two existing ones and the other one, which was unfortunate*. [R4]

The Eastern Seaboard Association of Tertiary Institutions (esATI), established in 2000, was a network of eight higher education institutions (HEIs) created in the apartheid era. It consisted of four universities: Durban-Westville, Natal, Zululand & University of South Africa together with four Technikons: Natal, ML Sultan, Mangosuthu and Technikon South Africa. The primary purpose of esATI was to offer joint public health postgraduate training across KwaZulu-Natal.

*So, ESATI was a consortium of universities. Initially, the dream was that ESATI would be offering the degree, but that dream never came true to the best of my knowledge… The reason for that was fairly clear: ESATI was a consortium. It was not a registered education institution, and therefore, ESATI could not award degrees. So, what happened was the way it worked in operational, practical terms was that different components of the MPH modules were offered at different universities.* [R1]

The esATI partnership could not be sustained due to several issues, including the fact that it involved different types of higher education institutions – technikons (now called technical universities) partnering with universities. This created tensions as the level of academic offering and exit outcomes differed between the different types of institutions. Since 2004, the newly formed University of KwaZulu-Natal has become the province’s sole provider of an MPH qualification (22).

*Some institutions were not always able to deliver; for instance, one institution offered an environmental health module, but they also found it quite difficult to deliver, as they had their own workloads. We were aiming for a high-level master’s, whereas they were much more laid-back, aiming for a lower-level master’s, where they [students] did not actually have to do a 50% research project*. [R5]

## Discussion

The development and expansion of postgraduate public health programmes in South Africa have been closely intertwined with the broader process of health sector reform following the end of apartheid in 1994. The transition to democracy created a demand for skilled public health professionals to support health system transformation and implement the health reforms (30, 31). The post-democratic government’s emphasis on primary health care, equity, and universal access required a workforce trained in epidemiology, health systems management, health promotion, environmental health, and health policy (28, 30). This also led to a shift from the historical dominance of physicians in population health to a more multidisciplinary approach in public health training. The establishment of MPH programmes across South African universities has contributed substantially to building a skilled public health workforce capable of supporting health system reform and improving population health outcomes (40–42).

One of the earliest policy frameworks guiding the transformation of the health sector in democratic South Africa was the White Paper for the Transformation of the Health System. It emphasized the need for reorientation of health professional training, advocating for a strong focus in training on population-based health planning, health promotion and education, and on the development of a foundational public health function (28). Implementing such reforms required professionals with expertise in population health and health service management, which is not widely taught in traditional clinical training (42, 43). Evidence suggests that public health graduates are utilising the skills acquired in the MPH programmes in the workplace, thereby contributing to the broader goals of health system transformation. However, the postgraduate public health qualification is not recognised as a requirement for senior public health leadership and management positions (40–42). Career pathways for public health graduates in the public sector remain poorly defined, with many health system roles prioritising clinical qualifications despite responsibilities related to population health management, policy development, and programme evaluation.

The Human Resource for Health (HRH) Strategy for South Africa, published in 2011, highlighted the need to establish public health units at district and provincial levels and called for MPH Health programmes to increase the output of graduates in order to ensure that the re-engineered primary health care approach is implemented as planned in order to improve population health outcomes (44). However, the career pathway of MPH graduates remains uneven due to the absence of a formal regulatory framework and varying levels of employer recognition. Addressing these challenges will require coordinated efforts between universities, government, and a national accrediting professional body to strengthen the institutional positioning of public health professionals within the national health workforce. Recognition is important in context of South Africa where clinical disciplines such as medicine, nursing, and other allied health professions have established professional councils, regulatory frameworks, and clear career trajectories.

The evolution of MPH programmes was also influenced by activism and leadership. The progressive health sector in South Africa has historically served as a pivotal force in promoting equity, justice, and transformation. Most of the pioneers of MPH programmes were part of the progressive movements and were activists that sought to challenge the racially segregated and inequitable health structures that defined the pre-1994 era. The progressive health sector movements, such as NAMDA and the Health Workers Society, played a critical and transformative role in bridging activism, academia, and policy (45, 46). Their efforts were grounded in principles of social accountability, community participation, and a commitment to addressing the social determinants of health, principles that continue to shape postgraduate public health training in South Africa (45, 46). However, the expansion of MPH programmes has unfolded amid a quadruple burden of disease, provincial fragmentation, uneven health system performance, and chronic under-investment in human resources for health.

Postgraduate public health programmes in South Africa have evolved since the early 90s, transitioning from narrowly focused medical training to multidisciplinary programmes designed to address complex population health challenges (22, 34, 41). This required a shift from a biomedical to a public health model, which views health as the product of interacting biological, social, economic, environmental, behavioural and health-system determinants. The formation of three regional schools of public health was conceived as a collaborative initiative among major tertiary institutions in KZN, Gauteng (formerly known as the Transvaal), and Cape Town (37). The initiative aimed to strengthen public health training capacity in the respective regions and sought to create an interinstitutional academic space that integrated teaching and research (37). Unfortunately, this could not be sustained due to institutional capacity constraints across universities as well as territorial and institutional bureaucracies. The territorial and institutional capacity continues to plague the postgraduate public health academic space. The institutional capacity across the universities remains an issue highlighted by an insufficient number of academics, which puts an extra burden on existing staff (22). In addition, the number of academics with doctorates is limited in some MPH programmes, which influences the “scholarship” in public health and public health training (22). Furthermore, regression has occurred in some institutions, where schools of public health have been reduced to departments due to institutional reorganisation, rationalisation, and insufficient prioritisation or recognition of the importance of public health. These regressive developments have the potential to influence recruitment, as senior scholars may prefer institutions where public health retains a distinct institutional identity, autonomy and leadership. Importantly, the institutional positioning of public health within universities has significant implications for teaching, research productivity, interdisciplinary collaboration, and policy influence. Independent schools of public health frequently have greater visibility and institutional leverage when seeking external funding and establishing international partnerships.

### Limitations

Although the key informants were instrumental in the evolution of postgraduate public health programmes, we only captured the views of six individuals, whose views could differ from their successors. Another limitation of this paper is that the scope of the document analysis was restricted to documents relating primarily to public health training and the development and provision of training opportunities. The study did not include a systematic review of individual public health curricula, including curriculum content, learning outcomes, teaching approaches, competency frameworks, assessment strategies, or the extent to which curricula are aligned with the evolving needs of the South African health system.

### Recommendations

Based on the study’s findings and limitations, future research should undertake a critical review and comparison of public health curricula across South African institutions to assess their alignment with national health priorities, public health competencies, workforce requirements, and emerging population health challenges. This would complement the findings of this paper and provide insight into the responsiveness of public health education in South Africa.

Secondly, the role and professional identity of postgraduate public health graduates should be more clearly defined within the South African health workforce. Establishing recognised occupational pathways, a clearly articulated scope of practice, and opportunities for career progression could improve the utilisation and retention of public health expertise within the health system.

Lastly, public health leaders should demonstrate the importance of the discipline of public health and advocate for dedicated government funding to universities in addition to student subsidies.

## Conclusion

The development of MPH programmes in South Africa was closely linked to national efforts to address inequities inherited from the apartheid health system and to build a multidisciplinary workforce capable of implementing population-based health strategies. The South African experience highlights both the opportunities and complexities of transforming public health education to ensure that it is socially responsive, contextually relevant, and aligned with the country’s evolving population health needs and health system priorities. Nevertheless, the lack of recognition and clear career pathways for MPH graduates remain an outstanding issue. Enhancing the formal recognition of postgraduate public health graduates will improve career opportunities for professionals and will also contribute to building a stronger, more resilient health system capable of responding to South Africa’s evolving population health needs. The South African experience demonstrates that contextually relevant and socially responsive MPH programmes are important for building public health capacity. At the same time, their impact depends on parallel efforts to create recognised professional pathways and strengthen institutional capacities and leadership.

## Data Availability

The datasets presented in this article are not readily available because consent to share data was not obtained from participants in this study. Requests to access the datasets should be directed to dlungwane@ukzn.ac.za.
