# Supplementary material for: Context and intricacies in the evolution of master of public health programmes in post-apartheid South Africa
Source: Front Public Health. 2026 Oct 14;14:1882277. doi: 10.3389/fpubh.2026.1882277 (PMC13713658; doi:10.3389/fpubh.2026.1882277)
Supplement: Supplementary file 1 [file Table_1.docx]

**List of documents that shaped the development and evolution of the MPH training programmes in South Africa**

| Author | Year | Title |
| --- | --- | --- |
| London L, Zweigenthal V, Pick W | 2022 | The Speciality of Public Health Medicine in South Africa: 1974–2021 |
| Dlungwane | 2017 | Master of Public Health programmes in South Africa: issues and challenges |
| Zweigenthal V, London L, Pick W | 2016 | The contribution of specialist training programmes to the development of a public health workforce in South Africa |
| Rispel L | 2016 | Analysing the progress and fault lines of health sector transformation in South Africa |
| van Rensburg HCJ. | 2014 | South Africa’s protracted struggle for equal distribution and equitable access – still not there. |
| Gear J | 2013 | Public health in South Africa 1975–89: reflections on a momentous past. |
| Pick W | 2013 | Reflections on Public Health in South Africa, 1993–2002. |
| Hoffman M, Coetzee D, Hodes R, London L | 2012 | From comprehensive medicine to public health at the University of Cape Town: a 40-year journey. |
| Ewert EG, Baldwin-Ragaven L, London L | 2011 | Training Trainers in health and human rights: Implementing curriculum change in South African health sciences institutions. |
| Alexander L, Igumbor E, Sanders D | 2009 | Building capacity without disrupting health services: public health education for Africa through distance learning. |
| Coovadia H, Jewkes R, Barron P, Sanders D, McIntyre D. | 2009 | The health and health system of South Africa: historical roots of current public health challenges |
| London L, Baldwin-Ragaven | 2008 | Human rights and health: Challenges for training nurses in South Africa. Curationis |
| Mokwena K, Mokgatle-Nthabu M, Madiba S, Lewis H, Ntuli-Ngcobo B | 2007 | Training of public health workforce at the National School of Public Health: meeting Africa's needs. |
| Republic of South Africa | 2003 | National Health Act |
| Reddy J | 1998 | Regional consortia, partnerships, mergers and their implications for transforming the South African higher education system. |
| Department of Health | 1997 | White Paper for the Transformation of the Health System in South Africa. |
| African National Congress | 1994 | The Reconstruction and Development Programme: A policy framework |
| Yach D, Tollman SM | 1993 | Public health initiatives in South Africa in the 1940s and 1950s: lessons for a post-apartheid era. |
